# Supplementary material for: A delayed fractionated dose RTS,S AS01 vaccine regimen mediates protection via improved T follicular helper and B cell responses
Source: eLife. 2020 Apr 29;9:e51889. doi: 10.7554/eLife.51889 (PMC7213985; doi:10.7554/eLife.51889)
Supplement: Supplementary file 1. — Supplementary file 1B. Parameters most predictive of protection using the early-response (pre-Dose 3) immune data. [file elife-51889-supp1.docx]

**Supplementary file 1A: Summary of immune measures**

| **Assay** | **T0** | **T1** | **T2** | **T3** | **T4** | **T5** | **T6** | **T7** |
| --- | --- | --- | --- | --- | --- | --- | --- | --- |
| AELI | 5 | 5 |  | 5 |  |  |  |  |
| BCF | 186 |  | 186 |  | 186 | 186 | 186 | 186 |
| BELI | 6 |  | 6 |  | 6 | 6 | 6 | 6 |
| ICC | 96 | 96 | 96 | 96 | 96 | 96 | 96 | 96 |
| IgG | 6 |  | 6 |  | 6 | 6 | 6 | 6 |
|  |  |  |  |  |  |  |  |  |

**Supplementary file 1A:** Spontaneous antibody secreting cell ELSIPOT (AELI), CSP- and HBs-specific B cell subsets by flow cytometry (BCF), frequencies and function of total pTfh, CSP-, HBs-and SEB-specific CD4 and pTfh data (Tfh ICC), CSP- and HBs-specific memory B cell ELISpot data (BELI), CSP-and HBs-specific PBMC culture supernatant IGG (IgG).

**Supplementary file 1B: Parameters most predictive of protection**

| **Cell Type** | **Phenotype** | **Parameter** | **Weight** |
| --- | --- | --- | --- |
| B cell | ELISPOT | BELI.CSP.T2 | 97 |
| T cell | CD40L+CD4 | CD40L.CSP.T2 | 100 |
|  |  | CD40L.CSP.T4 | 81 |
|  | Ag.pTfh | Ag.pTfh.IL.21.CSP.T4 | 46 |
|  |  | Ag.pTfh.CSP.T2 | 28 |
|  | Bulk Tfh | Bulk.Tfh.MED.T4 | 45 |
|  |  | Bulk.Tfh.MED.T2 | 32 |
|  | CD4 | CD4.SEB.T4 | 11 |
|  |  | CD4.CSP.T4 | 9 |
|  |  | CD4.HBs.T4 | 5 |

**Supplementary file 1B**: Parameters most predictive of protection using the early-response (pre-Dose 3) immune data
