# Supplementary material for: A delayed fractionated dose RTS,S AS01 vaccine regimen mediates protection via improved T follicular helper and B cell responses
Source: eLife. 2020 Apr 29;9:e51889. doi: 10.7554/eLife.51889 (PMC7213985; doi:10.7554/eLife.51889)
Supplement: Transparent reporting form [file elife-51889-transrepform.docx]

***eLife’s* transparent reporting form**

We encourage authors to provide detailed information *within their submission* to facilitate the interpretation and replication of experiments. Authors can upload supporting documentation to indicate the use of appropriate reporting guidelines for health-related research (see [EQUATOR Network](http://www.equator-network.org/%20)), life science research (see the [BioSharing Information Resource](https://biosharing.org/)), or the [ARRIVE guidelines](http://www.plosbiology.org/article/info:doi/10.1371/journal.pbio.1000412) for reporting work involving animal research. Where applicable, authors should refer to any relevant reporting standards documents in this form.

If you have any questions, please consult our Journal Policies and/or contact us: [editorial@elifesciences.org](mailto:editorial@elifesciences.org).

**Sample-size estimation**

- You should state whether an appropriate sample size was computed when the study was being designed
- You should state the statistical method of sample size computation and any required assumptions
- If no explicit power analysis was used, you should describe how you decided what sample (replicate) size (number) to use

Please outline where this information can be found within the submission (e.g., sections or figure legends), or explain why this information doesn’t apply to your submission:

We did not use sample size estimation analysis in this study as this was conducted as an immunology sub-study of a clinical trial NCT91857869. This clinical trials is a phase 2a, controlled, open-label, study of healthy malaria-naive adults, 16 subjects vaccinated with a 0-, 1-, and 2-month full-dose regimen (012M) and 30 subjects who received a 0-, 1-, and 7-month regimen, including a fractional third dose (Fx017M), underwent CHMI 3 weeks after the last dose. We used deidentified samples that are already collected at different timepoints for this substudy.

**Replicates**

- You should report how often each experiment was performed
- You should include a definition of biological versus technical replication
- The data obtained should be provided and sufficient information should be provided to indicate the number of independent biological and/or technical replicates
- If you encountered any outliers, you should describe how these were handled
- Criteria for exclusion/inclusion of data should be clearly stated
- High-throughput sequence data should be uploaded before submission, with a private link for reviewers provided (these are available from both GEO and ArrayExpress)

Please outline where this information can be found within the submission (e.g., sections or figure legends), or explain why this information doesn’t apply to your submission:

An assay optimization phase was conducted before the study using cells obtained from a previous malaria vaccine study by GSK. We optimized antigen (Ag) concentrations required for each assay and established the analysis strategy for the main study. For the current study, samples were processed longitudinally for each participant to minimize the assay variation and technical errors. For stimulation experiments, an unstimulated condition was used as biological controls. For flow cytometry analysis, all the antibodies were titrated for the optimal concentration. Proper instrument calibration and fluorescence compensation were used for each experiment. For the gating purpose to identify the population of interest, unstained control and a gating control using basic phenotypic subset markers were included. We did not exclude any outliers in this study.

**Statistical reporting**

- Statistical analysis methods should be described and justified
- Raw data should be presented in figures whenever informative to do so (typically when N per group is less than 10)
- For each experiment, you should identify the statistical tests used, exact values of N, definitions of center, methods of multiple test correction, and dispersion and precision measures (e.g., mean, median, SD, SEM, confidence intervals; and, for the major substantive results, a measure of effect size (e.g., Pearson's r, Cohen's d)
- Report exact p-values wherever possible alongside the summary statistics and 95% confidence intervals. These should be reported for all key questions and not only when the p-value is less than 0.05.

Please outline where this information can be found within the submission (e.g., sections or figure legends), or explain why this information doesn’t apply to your submission:

Statistical method used for this study has been extensively elaborated in the Materials and Methods section with a subtitle entitled “Statistical analysis and data integration”. Fig 2-5, used a Fit generalized linear mixed-effects model (GLMM) via Penalized Quasi-Likelihood (PQL) using R “MASS” package to accommodate repeated measure of time, with random intercept set by participant. P values shown within the graph refer to significant difference between Protected and Non-Protected groups indicated time points. Fig 6-7 used machine-learning methods using random forest model.

(For large datasets, or papers with a very large number of statistical tests, you may upload a single table file with tests, Ns, etc., with reference to sections in the manuscript.)

**Group allocation**

- Indicate how samples were allocated into experimental groups (in the case of clinical studies, please specify allocation to treatment method); if randomization was used, please also state if restricted randomization was applied
- Indicate if masking was used during group allocation, data collection and/or data analysis

Please outline where this information can be found within the submission (e.g., sections or figure legends), or explain why this information doesn’t apply to your submission:

All lab testing, analysis, data entry and plotting of graphs were performed in batches in a blinded manner in the University of Miami lab. The protection status was disclosed once the data-base had been locked. Vaccine arm identity, Ab responses were revealed to the UM lab after all samples had been processed.

**Additional data files (“source data”)**

- We encourage you to upload relevant additional data files, such as numerical data that are represented as a graph in a figure, or as a summary table
- Where provided, these should be in the most useful format, and they can be uploaded as “Source data” files linked to a main figure or table
- Include model definition files including the full list of parameters used
- Include code used for data analysis (e.g., R, MatLab)
- Avoid stating that data files are “available upon request”

Please indicate the figures or tables for which source data files have been provided:

Source data file for Figure 2-5, figure 5-figure supplement 2-5 are included as an excel file. Complete database corresponding to the work and study presented here is available upon request at GSK
